# Long-Term Retrospective Predicted Concentration of PM_2.5_ in Upper Northern Thailand Using Machine Learning Models

**DOI:** 10.3390/toxics13030170

**Published:** 2025-02-27

**Authors:** Sawaeng Kawichai, Patumrat Sripan, Amaraporn Rerkasem, Kittipan Rerkasem, Worawut Srisukkham

**Affiliations:** 1 Research Institute for Health Sciences, Chiang Mai University, Chiang Mai 50200, Thailand; sawaeng.kaw@cmu.ac.th (S.K.); patumrat.sripan@cmu.ac.th (P.S.); amaraporn.rer@cmu.ac.th (A.R.); 2 Clinical Surgical Research Center, Department of Surgery, Faculty of Medicine, Chiang Mai University, Chiang Mai 50200, Thailand; 3 Department of Computer Science, Faculty of Science, Chiang Mai University, 239 Huay-Kaew Road, Suthep, Muang, Chiang Mai 50200, Thailand

**Keywords:** PM_2.5_ prediction, retrospective prediction, long-term prediction, machine learning, fire hotspots

## Abstract

This study aims to build, for the first time, a model that uses a machine learning (ML) approach to predict long-term retrospective PM_2.5_ concentrations in upper northern Thailand, a region impacted by biomass burning and transboundary pollution. The dataset includes PM_10_ levels, fire hotspots, and critical meteorological data from 1 January 2011 to 31 December 2020. ML techniques, namely multi-layer perceptron neural network (MLP), support vector machine (SVM), multiple linear regression (MLR), decision tree (DT), and random forests (RF), were used to construct the prediction models. The best ML prediction model was selected considering root mean square error (RMSE), mean prediction error (MPE), relative prediction error (RPE) (the lower, the better), and coefficient of determination (R^2^) (the bigger, the better). Our study found that the ML model-based RF technique using PM_10_, CO_2_, O_3_, fire hotspots, air pressure, rainfall, relative humidity, temperature, wind direction, and wind speed performs the best when predicting the concentration of PM_2.5_ with an RMSE of 6.82 µg/m^3^, MPE of 4.33 µg/m^3^, RPE of 22.50%, and R^2^ of 0.93. The RF prediction model of PM_2.5_ used in this research could support further studies of the long-term effects of PM_2.5_ concentration on human health and related issues.

## 1. Introduction

Air pollution has been an issue in upper northern Thailand for several years, and the haze situation that has been caused by forest fires is a significant factor in this problem [1,2,3,4]. This burning causes a pollution problem throughout the dry season, which typically lasts from the end of February to the middle of April [1,2,3,4]. It is likely that exposure to specific environmental pollutants could have long-term effects and risk factors that contribute to an increased probability of sustaining lung cancer [5]. During this period, the amount of particulate matter smaller than 10 and 2.5 microns (PM_10_ and PM_2.5_) in the atmosphere exceeds the standards of Thailand. Moreover, the PM_2.5_ concentrations measured during the sampling period of 24 h exceeded the PM_2.5_ Thailand Ambient Air Quality Standard (50 µg/m^3^) by less than 30.60% (112 days in 2019) [6].

In this region, there is a significant gap in research on the long-term health effects of PM_2.5_ exposure, primarily due to the lack of comprehensive, long-term retrospective data on PM_2.5_ concentrations. The absence of data complicates the understanding of the impact of long-term air pollution exposure on health, particularly on lung cancer, cardiovascular diseases (CVDs), and chronic obstructive pulmonary disease (COPD) [5]. The lack of comprehensive long-term datasets limits the development of reliable evidence-based public health policies. In Chiang Mai, upper northern Thailand, PM_2.5_ monitoring was started in 2011, while PM_2.5_ data later became available in Lampang in 2018 and in other provinces in 2019, including Chiang Rai, Lamphun, Phayao, Phrae, Nan, and Mae Hong Son, owing to the limitations of resources. The importance of this work extends beyond regional limits, as the modeling methodology may be modified for application in other areas facing comparable problems with air quality. The combination of multiple sources of data, such as air pollutant concentrations, fire hotspot information, and meteorological variables, provides a thorough methodology for fulfilling the essential requirement for historical PM_2.5_ data in environmental health research. In upper northern Thailand, PM_10_ has been widely monitored for more than 20 years [7]. Predicting the results of PM_2.5_ values using PM_10_ [8,9,10] and fire hotspot data [11,12] with critical meteorological data [13,14] allows the study of the long-term effects of past exposure to PM_2.5_ on various health problems.

In previous studies, predictive methods have used multivariate statistical analysis, but in the last two decades, artificial intelligence technology using machine learning (ML) has been applied to create a model for forecasting or predicting air quality with an ability to predict results that are better than operational air quality measurements [15,16]. As a result, a wide range of research studies have been conducted that have applied various machine learning techniques such as artificial neural networks (ANNs) and support vector machines. Random forests classification has also been used to create a model for air quality prediction [15,16]. Studies on the model for predicting PM_2.5_ and PM_10_ concentrations revealed that various machine learning algorithms, capable of managing intricate and non-linear relationships among air quality variables, can effectively predict the value of new, unseen data with remarkable efficiency and precision [15,16,17,18,19]. ANNs are techniques for machine learning that mimic the neural activity of the human brain, appearing like nodes arranged in one or more layers. The nodes communicate with each other and store information in the form of the weight of each line connecting the nodes. This technique can retain knowledge that it has acquired and has been used in many tasks, including pattern recognition, bioinformatics, prediction, and other applications in many fields [19]. MLP neural networks are also widely used in predictive modeling. ML models provide highly accurate prediction results for PM_2.5_ and PM_10_ dust content [15,17,18,19]. This is the first time a model has been built that uses an ML approach to predict long-term retrospective PM_2.5_ concentrations in upper northern Thailand, a region impacted by biomass burning and transboundary pollution. The modeling framework developed here could not only be applied to northern Thailand, but also adapted to other regions with similar air quality issues. Furthermore, the retrospective study of PM_2.5_ data usually encounters spatial and temporal limitations due to the absence of government-provided monitoring stations for PM_2.5_ measurement in upper northern Thailand over the past decade. Therefore, several critical factors warrant the development of an ML model to predict retrospective PM_2.5_. Additionally, this integrative strategy not only bridges gaps in direct monitoring, but also advances our understanding of how exposure to these environmental factors could have long-term effects and risk factors that cause health issues. The main objective of this study is to apply ML methods to create a model for predicting retrospective PM_2.5_ values using air pollutant concentrations, fire hotspot data, and meteorological data.

## 2. Materials and Methods

### 2.1. Descriptions of the Data

**Figure 1:**
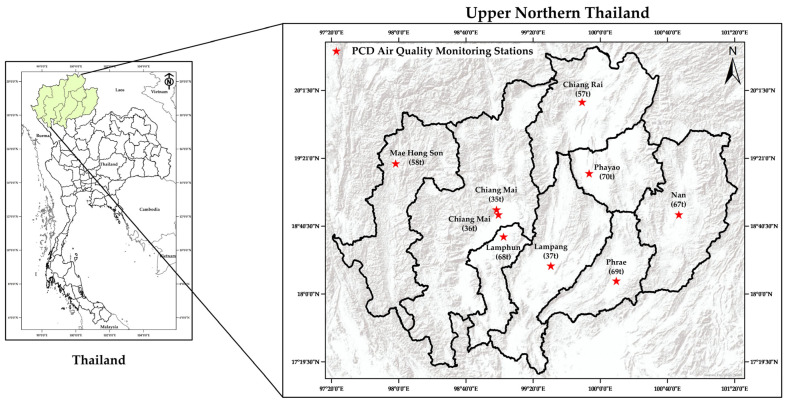
The Pollution Control Department (PCD)’s monitoring stations in upper northern Thailand.

Air pollutant concentrations including PM_2.5_, PM_10_, CO_2_, SO_2_, NO_2_, and O_3_; fire hotspot data; and critical meteorological data including air pressure, rainfall, relative humidity, temperature, wind direction, and wind speed from 1 January 2011 to 31 December 2020 were collected from eight of the upper northern provinces of Thailand—Chiang Mai, Lampang, Chiang Rai, Lamphun, Phayao, Phrae, Nan, and Mae Hong Son—using the official database of the Pollution Control Department (PCD). In the PCD’s monitoring station, PM_2.5_ and PM_10_ concentrations were measured via the tapered element oscillating microbalance method (TEOM) and then averaged at the data center to produce a time series of the daily mean of air pollutant concentrations and meteorological data. Air quality and meteorological data were collected as daily mean values from fixed monitoring stations operated by the PCD in each province. All datasets were aggregated on a daily basis and assigned a location number, e.g., 35t and 36t represent Chiang Mai province. This synchronization ensured that the model used co-located information from all data sources for each day. The PCD’s monitoring stations, located in eight of the provinces of upper northern Thailand, are shown in Figure 1.

The daily fire hotspot number was retrieved from NASA’s Fire Information for Resource Management System (FIRMS). In this research, we obtained fire hotspot data from the MODIS Terra and Aqua Collection 6.1 via the NASA Level-1 and atmospheric archive and distribution system [20].

### 2.2. Predictive Model

The PM_2.5_ prediction models were constructed by employing twelve input parameters: (1) PM_10_, (2) CO_2_, (3) SO_2_, (4) NO_2_, (5) O_3_, (6) fire hotspots, (7) air pressure, (8) rainfall, (9) relative humidity, (10) temperature, (11) wind direction, and (12) wind speed. The PM_2.5_ data were collected during different time periods for each province—in 2011 for Chiang Mai, 2018 for Lampang, and 2019 for Chiang Rai, Lamphun, Phayao, Phrae, Nan, and Mae Hong Son. The SO_2_ data were not available for Mae Hong Son province, and the NO_2_ data in this province were incomplete and inconsistent. So, we built the predictive model of PM_2.5_ based on the data from Chiang Mai province, which has the oldest, longest, and most complete air quality and meteorological data from two monitoring stations (Figure 1).

Among 6974 records from Chiang Mai province used as training data, the amounts of missing data were 0.06% for NO_2_, 0.20% for SO_2_, 0.68% for rainfall, 1.15% for CO_2_, 1.15% for O_3_, and 47.57% for air pressure. We compared the performance of the model when using different numbers of features: (1) all 12 input features, (2) 11 input features (without SO_2_), and (3) 10 input features (without SO_2_ and NO_2_).

The predictive models were built using supervised ML. The models were trained on a labeled dataset, meaning that each input data point had a corresponding output, which was the PM_2.5_ concentration. The goal was for the model to learn the relationship between each input feature and the output so that it could predict the output for unseen data. One of the ML techniques used in this study was multi-layer perceptron (MLP), which is one of the most popular supervised neural network modelling techniques. It has been widely used in pattern recognition, bioinformatics, and computer vision and control systems [21]. MLP is a modern feed-forward neural network that consists of fully connected neurons or nodes. The nodes have a non-linear activation function, which is responsible for processing and giving answers to the next connecting nodes. It is usually trained using the backpropagation algorithm. Additionally, MLP consists of at least three layers of node networks: an input layer, a hidden layer, and an output layer [22]. In this study, we constructed MLP models using both a single hidden layer and two hidden layers. We employed the Levenberg–Marquardt backpropagation learning technique for both MLP models. We employed the Sigmoid activation function for the hidden layer and the Linear activation function for the output layer in a single hidden-layer configuration. The learning rate was 0.1, the momentum rate was 0.8, and the number of nodes varied from 1 to 50. Furthermore, for the MLP with two hidden layers, we employed the Sigmoid activation function for the hidden levels and the Linear activation function for the output layer. The learning rate was set at 0.1 and the momentum rate at 0.8, and the quantity of hidden-layer nodes varied from 1 to 30. We also used support vector machine (SVM), which is a kernel-based classification method. In general, it has to compute a linear function in a higher dimensional feature space, where the lower dimensional input data are mapped using a kernel function. It is used extensively in many fields such as prediction, pattern recognition, and classification [23]. This study involved constructing SVM models that utilize three distinct kernels: Linear, Polynomial, and Radial Basis functions. We employed grid search as the optimization method. The maximum objective evaluation and the maximum iteration were both set at 100. Multiple linear regression (MLR), which we also used, is a statistical model that estimates the relationships between one dependent variable and more independent variables by fitting multiple lines to the observed data. MLR extends a simple linear regression to include more than one explanatory variable to predict the outcome of a response variable [24,25]. Moreover, it is a widely used technique in many fields such as social sciences research, econometrics, and financial inference. In this study, we constructed the MLR model by using the “regress()” function for the multiple linear regression with a 95% confidence interval and setting epsilon (ε) to 0. Another technique we used was decision tree (DT), which is one of the general-purpose computationally intensive statistical algorithms for prediction and classification, artificial intelligence, machine learning, and knowledge discovery. DT has to do with using a procedure or rule repeatedly to generate subsetting of the target subject of data according to the values of associated input subjects to make partitions, and associated descendent leaves or nodes of the tree, that contain progressively similar intra-node target values and progressively dissimilar inter-node values at any given height of the tree [26]. This study involved the construction of a DT regression model utilizing a grid search optimizer, with the objective evaluation maximum set as 30. Random forests (RF) is another technique we used and is one of the famous ensemble machine learning techniques. Researchers have widely used it due to its good performance and simple usage [27,28]. This technique uses multiple decision trees. The trees’ predictors are taught using random sample data, and the distribution is the same for the predictors of all trees in the forest. The primary voting method is used to choose the greatest number of identical answers (majority voting). In this study, we developed an RF model with the following hyperparameters: the number of trees was set as 10, the maximum depth of the trees was set as 5, and the number of learning cycles for the trees was set as 200. The experiments in this work were performed using MATLAB R2018a. A summary of the hyperparameter settings for the machine learning models for the dataset from Chiang Mai province (air quality data, meteorological data, and fire hotspots) is shown in Table S1.

### 2.3. Model Validation

The best ML prediction model was selected considering the root mean square error (RMSE), mean prediction error (MPE), relative prediction error (RPE) (the lower, the better), and coefficient of determination (R^2^) (the bigger, the better) [29,30,31]. Additionally, in the evaluation of each run, 10-fold cross-validation [32] was implemented, dividing the dataset into 10 equally sized folds. In each iteration, 1 fold was designated as the validation set, with the remaining 9 folds used for training. This procedure was repeated until every fold had been used as the validation set once. This validation was applied to the dataset, which was divided into 70% for training and 30% for testing.

### 2.4. Prediction Evaluation Visual Check

The predicted PM_2.5_ was compared with the observed data to evaluate the performance of the model. Visual inspection was used to confirm that the predicted and observed data were aligned as their two-way plots were on the identical line. The RMSE, R^2^, MPE, and RPE were provided in addition to the graphical check. We performed the evaluation for both seen data (data for training model) and unseen data (data for testing model). This research used data from Chiang Mai province, which has the oldest, longest, and most complete data, for the visual check of the training data. Additionally, data from eight provinces were employed for the visual check of the unseen data.

## 3. Results

The air quality data, meteorological data, and data on fire hotspots in Chiang Mai province were separated into two parts, a dataset for training and a dataset for testing. The performances of the ML models—MLP, SVM, MLR, DT, and RF—are shown in Table 1.

When using all features, the RF model is the best model, considering its lowest RMSE at 6.7615 µg/m^3^, MPE at 4.1954 µg/m^3^, RPE at 22.29%, and highest R^2^ at 0.9318. Therefore, the RF model was selected and used for the next steps of this study.

### 3.1. Performance of RF Model with Different Features

Based on the dataset from Chiang Mai province, after removing SO_2_, the RMSE, MPE, and RPE slightly increased to 6.8234 µg/m^3^, 4.2499 µg/m^3^, and 22.49%, respectively, and R^2^ was reduced to 0.9306. The performance of the model without SO_2_ was not different when compared to the full features model (*p* > 0.05). When NO_2_ was removed from the reduced model, the RMSE, MPE, and RPE increased to 6.8242 µg/m^3^, 4.3296 µg/m^3^, and 22.50%, respectively, while the R^2^ did not change. No significant change in model performance was observed when the number of features was reduced to 10. The performance of the model without SO_2_ and NO_2_ was not different when compared to the full model (*p* > 0.05) (Table 2).

### 3.2. Performance of RF During PM_2.5_ Prediction in Eight Provinces in Upper Northern Thailand

**Figure 2:**
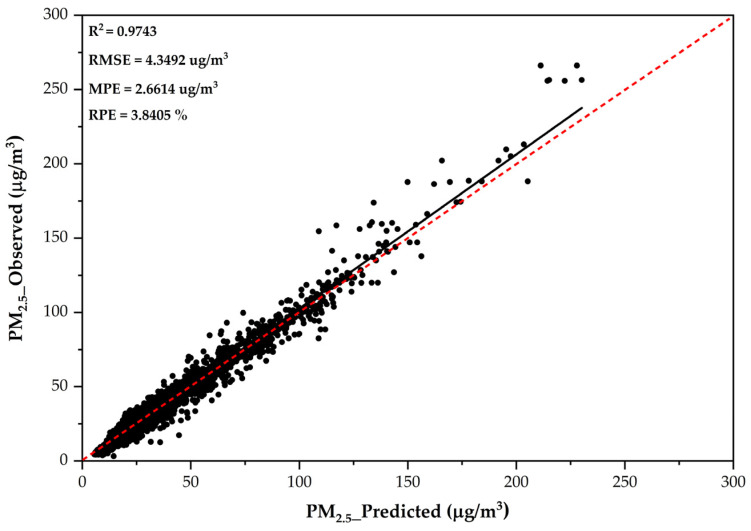
The performance of the RF model during PM_2.5_ prediction using training data from Chiang Mai province.

The predictive model without SO_2_ and NO_2_ was used to predict PM_2.5_ in eight provinces in Northern Thailand. Figure 2 demonstrates the performance of the RF model on the training data, employing data from Chiang Mai province.

**Figure 3:**
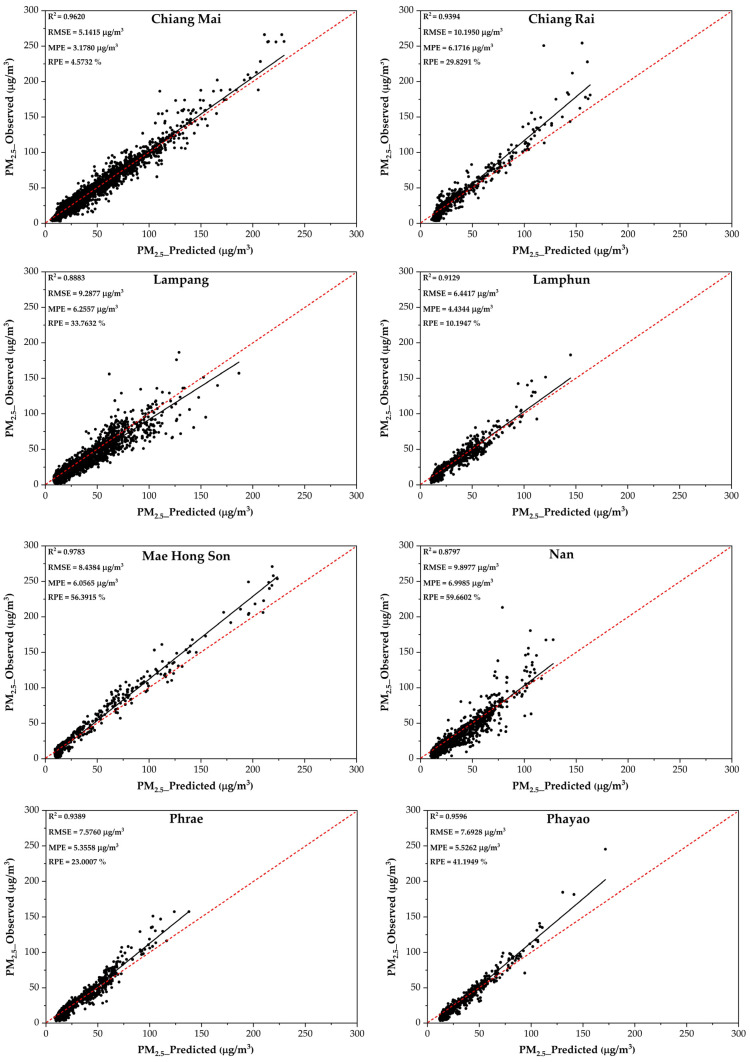
The performance of the RF model during PM_2.5_ prediction using testing data from eight provinces in upper northern Thailand.

The value of R^2^ for the model used is 0.9743, while the R^2^ ranged from 0.8797 to 0.9783 for the testing data (Figure 3). The R^2^ was highest in Mae Hong Son province and lowest in Nan province. The performance of the model considering RMSE, MPE, and RPE indicated the same direction.

## 4. Discussion

From this study, it was found that the RF model was the most effective and had the highest accuracy in predicting PM_2.5_ concentrations compared to the other models. This is consistent with a study by Chen [33] that predicted PM_2.5_ using eight types of air quality data, as well as five types of meteorological data. Chen’s study found that using the RF model was the most efficient way to predict PM_2.5_, which had a relatively high R^2^ value of 0.94. In 2023, Vignesh et al. performed a study that employed ML techniques. There were nine models, and a tool was established to evaluate their performance and accuracy when predicting PM_2.5_ concentrations. The research, which was conducted in the United States, employed air pollution data collected over a period of five years, from 2017 to 2021 [34]. The investigation revealed that the RF model demonstrated high effectiveness when predicting concentrations of PM_2.5_, with an R^2^ of 0.77.

The higher concentrations of particulate pollution observed during the dry season are most likely a result of significant biomass burning, specifically from agricultural activities performed in preparation for the next agricultural season. Another issue is the transboundary transport of air pollution originating from neighboring countries such as Laos, Vietnam, and Myanmar, which is influenced by meteorological situations. These elements increase the problem of air quality in upper northern Thailand [35,36]. This study built a prediction model with data from Chiang Mai province. The unique model can be applied to eight provinces because of their comparable area characteristics. The primary factors contributing to air pollution in northern Thailand include biomass combustion, meteorological conditions, and geographical characteristics [37]. Moreover, a wide range of parameters have a significant impact on R^2^, with geographical data being particularly important. Various variables influence the value of R^2^, with Mae Hong Son province having the most significant influence. Mae Hong Son is a small province surrounded by forest. Mae Hong Son is located close to the Thailand–Myanmar border. Mae Hong Son province experiences multiple sources of air pollution, including transboundary effects, forest fires, and biomass burning, as indicated in prior research conducted by Kliengchuay et al. [38]. Forest fires play an important part in the emission of PM_2.5_ in this area. The complicated relationships between meteorological conditions and PM_2.5_ levels affect the numerous relations between PM_2.5_ and meteorology [39], while the lower R^2^ in an area like Nan, which is a large province, may be influenced by various meteorological parameters such as rainfall, wind direction, wind speed, temperature, relative humidity, and air pressure. Moreover, the lack of clarity regarding development across large areas could be contributing to the lower R^2^ [40,41].

Additionally, this study shows that the RF model is the most effective in predicting PM_2.5_, consistent with the findings of research conducted by Chen et al. [33] and Vignesh et al. [34]. However, our study differs from those studies in that we have included fire hotspots as a feature in our ML model for PM_2.5_ prediction, in addition to factors such as the environment, the climate, and geographical characteristics. The topography, meteorological data, and agriculture of Southeast Asia significantly contributes to the prevalence of monoculture farming, resulting in an important number of fire hotspots. These regions in Southeast Asia show evidence of biomass burning, which emits PM_2.5_ pollutants [1,2,3,4]. Thus, our study employs the number of fire hotspots as one of the important features for modeling the PM_2.5_ predictor.

The retrospective PM_2.5_ data from our research could be helpful for studying the long-term effects of PM_2.5_ concentrations on human health issues such as lung cancer, cardiovascular diseases (CVDs), and chronic obstructive pulmonary disease (COPD). There are relatively few studies on the impact of exposure to PM_2.5_ on lung cancer incidence rates among Asian populations. There are literature review studies that aim to explore the relationship between PM_2.5_ and lung cancer incidence and mortality. One review study shows that some studies have found a significant relationship between PM_2.5_ and the incidence of lung cancer, but other studies did not find this relationship [5]. The limited number of studies on the impact of exposure to PM_2.5_ on lung cancer may be caused by the limited long-term PM_2.5_ data, particularly in low- and middle-income countries. Our predicted PM_2.5_ data, as shown in Supplementary Table S2, could be included in epidemiological health outcome prediction models to clarify PM_2.5_-related health risks in upper northern Thailand. Additionally, investigating the relationships between socio-economic characteristics, healthcare accessibility, and health outcomes associated with PM_2.5_ would enhance comprehension of the differences in disease burden. Long-term cohort studies that monitor individuals over time, considering both environmental exposures and personal health data, could be essential for enhancing exposure response models and guiding public health strategies to reduce the harmful effects of air pollution. Finally, the PM_2.5_ concentration values from the past 10 years (2011 to 2020) that were predicted during our study could be used to investigate the long-term impact of PM_2.5_ on acute and chronic respiratory diseases, as well as to study other health-related effects of PM_2.5_ in eight provinces in the upper northern region of Thailand, an area where PM_2.5_ concentration levels are reported to exceed Thailand’s ambient air quality standard every year.

## 5. Conclusions

This study found that the RF model was the most effective in predicting PM_2.5_ concentrations, outperforming other models in terms of accuracy. It also highlights the significant impact of biomass burning and fire hotspots on the prediction of PM_2.5_ concentrations. Our study illustrates the significance of employing numerous data sources and effective methods for modeling in environmental studies. Moreover, future work could apply the RF model, which is a highly effective tool for predicting long-term PM_2.5_ concentrations (RMSE of 6.82 μg/m^3^, MPE of 4.33 μg/m^3^, RPE of 22.50%, and R^2^ of 0.93), to other regions or countries with similar environmental conditions, including biomass burning and transboundary pollution. The predicted PM_2.5_ concentrations could lead to improved air quality management strategies and more informed public health policies. Furthermore, our research suggests that the prediction model of prolonged PM_2.5_ concentrations could offer a foundation for further epidemiological studies on the long-term effects of PM_2.5_ concentrations on human health and related problems.

## Figures and Tables

**Table 1:** Performances of ML models for PM_2.5_ prediction using different numbers of features.

Methods	Prediction Performances											
	10 Features (Without SO_2_ and NO_2_)				11 Features (Without SO_2_)				12 Features			
	RMSE	R^2^	MPE	RPE	RMSE	R^2^	MPE	RPE	RMSE	R^2^	MPE	RPE
MLP (1 Hidden Layer)	7.2287	0.9211	4.7944	23.88	7.2136	0.9214	4.8845	23.84	7.1802	0.9221	4.8121	23.73
MLP (2 Hidden Layers)	7.3328	0.9181	4.8184	24.24	7.3265	0.9189	4.8822	24.19	7.2367	0.9210	4.8854	23.91
SVM (Linear Kernel)	10.7402	0.8223	8.2057	35.51	11.1608	0.8111	8.4791	36.79	11.7684	0.7752	9.2530	38.99
SVM (Polynomial Kernel)	12.9420	0.7367	10.2391	42.70	12.5287	0.7602	9.8174	41.38	12.1642	0.7621	8.9826	40.08
SVM (RBF Kernel)	12.0770	0.7748	8.9261	39.91	12.6847	0.7539	9.6007	41.82	12.1247	0.7755	9.0067	40.01
MLR	7.7423	0.9103	5.2223	25.55	7.7415	0.9103	5.2270	25.55	7.7056	0.9111	5.2141	25.44
DT	9.0747	0.8762	5.8224	29.96	8.7843	0.8840	5.5989	29.01	8.9378	0.8800	5.6141	29.52
RF	6.8242	0.9306	4.3296	22.50	6.8234	0.9306	4.2499	22.49	6.7615	0.9318	4.1954	22.29

Note: RMSE: root mean square error (µg/m^3^); R^2^: coefficient of determination; MPE: mean prediction error (µg/m^3^); RPE: relative prediction error (%).

**Table 2:** Comparison between PM_2.5_ prediction performances of RF model with different features.

Performances	12 Features	11 Features	*p*-Value	10 Features	*p*-Value
Average RMSE	6.7859	6.8110	0.8798	6.8216	0.9397
Average R^2^	0.9313	0.9308	0.9397	0.9307	0.8798
Average MPE	4.3290	4.2533	0.2568	4.1944	0.3258
Average RPE	22.3740	22.4551	0.9397	22.4884	1.0000

## Data Availability

The datasets used and/or analyzed during the current study are available from the corresponding author upon reasonable request.

## Supplementary Materials

The following supporting information can be downloaded at: https://www.mdpi.com/article/10.3390/toxics13030170/s1, Table S1: The hyperparameters of the machine learning models for the dataset from Chiang Mai province (air quality data, meteorological data, and fire hotspots); Table S2: The predicted PM_2.5_ concentrations in 8 provinces in northern Thailand, 2011–2020.

## Abbreviations

The following abbreviations are used in this manuscript:

ANNs	Artificial neural networks
COPD	Chronic obstructive pulmonary disease
CVDs	Cardiovascular diseases
DT	Decision tree
FIRMS	Fire Information for Resource Management System
ML	Machine learning
MLP	Multi-layer perceptron neural network
MLR	Multiple linear regression
MODIS	Moderate Resolution Imaging Spectroradiometer
NASA	National Aeronautics and Space Administration
PCD	Pollution Control Department
PM_2.5_	Particulate matter smaller than 2.5 microns
PM_10_	Particulate matter smaller than 10 microns
RF	Random forests
SVM	Support vector machine
TEOM	Tapered element oscillating microbalance method
