# Supplementary material for: Long-Term Retrospective Predicted Concentration of PM2.5 in Upper Northern Thailand Using Machine Learning Models
Source: Toxics. 2025 Feb 27;13(3):170. doi: 10.3390/toxics13030170 (PMC11946178; doi:10.3390/toxics13030170)
Supplement: Supplementary file 1 [file toxics-13-00170-s001.zip › toxics-3462418_Supplementary Table S1.pdf]

Table S1. Hyperparameters of the machine learning models for the dataset of Chiang Mai province (air quality data, meteorological data, and fire hotspots)

**Model**	**Details**
Multilayers-perceptron (MLP :1 Hidden Layer)	Multilayers-perceptron one hidden-layer with Levenberg-Marquardt backpropagation learning algorithm, activation function for hidden-layer is Sigmoid, activation function for output-layer is Linear, learning rate is 0.1, momentum rate is 0.8, and number of nodes range from 1 to 50 nodes.
Multilayers-perceptron (MLP: 2 Hidden Layers)	Multilayers-perceptron two hidden-layers with Levenberg-Marquardt backpropagation learning algorithm, activation function for hidden-layer is Sigmoid, activation function for output-layer is Linear, learning rate is 0.1, momentum rate is 0.8, and each hidden-layer number of nodes range from 1 to 30 nodes.
Support Vector Machine (SVM: Linear Kernel)	Kernel function is Linear Function, Optimizer is grid-search, Maximum Objective Evaluation is 100, and Maxmum Iteration is 100.
Support Vector Machine (SVM: Polynomial Kernel)	Kernel function is Polynomial Function, Optimizer is grid-search, Maximum Objective Evaluation is 100, and Maxmum Iteration is 100.
Support Vector Machine (SVM: RBF Kernel)	Kernel function is Radial Basis Function, Optimizer is grid-search, Maximum Objective Evaluation is 100, and Maxmum Iteration is 100.
Multiple Linear Regression (MLR)	Using function “Regress()” for the Multiple Linear Regression with 95% confidence interval, and epsilon (ε) is 0.
Decision tree (DT)	Decision tree regression with grid search optimizer, Maximum of Objective Evaluation is 30.
Random Forest (RF)	Number of trees is 10, Maximum depth of the trees is 5, and Number of trees learning cycles is 200.
